# Aesthetic Responses to Exact Fractals Driven by Physical Complexity

**DOI:** 10.3389/fnhum.2016.00210

**Published:** 2016-05-20

**Authors:** Alexander J. Bies, Daryn R. Blanc-Goldhammer, Cooper R. Boydston, Richard P. Taylor, Margaret E. Sereno

**Affiliations:** ^1^Visual Neuroscience Laboratory, Department of Psychology, University of OregonEugene, OR, USA; ^2^Fractals Research Laboratory, Department of Physics, University of OregonEugene, OR, USA

**Keywords:** aesthetics, complexity, fractal dimension, order, preference, recursion, symmetry

## Abstract

Fractals are physically complex due to their repetition of patterns at multiple size scales. Whereas the statistical characteristics of the patterns repeat for fractals found in natural objects, computers can generate patterns that repeat exactly. Are these exact fractals processed differently, visually and aesthetically, than their statistical counterparts? We investigated the human aesthetic response to the complexity of exact fractals by manipulating fractal dimensionality, symmetry, recursion, and the number of segments in the generator. Across two studies, a variety of fractal patterns were visually presented to human participants to determine the typical response to exact fractals. In the first study, we found that preference ratings for exact midpoint displacement fractals can be described by a linear trend with preference increasing as fractal dimension increases. For the majority of individuals, preference increased with dimension. We replicated these results for other exact fractal patterns in a second study. In the second study, we also tested the effects of symmetry and recursion by presenting asymmetric dragon fractals, symmetric dragon fractals, and Sierpinski carpets and Koch snowflakes, which have radial and mirror symmetry. We found a strong interaction among recursion, symmetry and fractal dimension. Specifically, at low levels of recursion, the presence of symmetry was enough to drive high preference ratings for patterns with moderate to high levels of fractal dimension. Most individuals required a much higher level of recursion to recover this level of preference in a pattern that lacked mirror or radial symmetry, while others were less discriminating. This suggests that exact fractals are processed differently than their statistical counterparts. We propose a set of four factors that influence complexity and preference judgments in fractals that may extend to other patterns: fractal dimension, recursion, symmetry and the number of segments in a pattern. Conceptualizations such as Berlyne’s and Redies’ theories of aesthetics also provide a suitable framework for interpretation of our data with respect to the individual differences that we detect. Future studies that incorporate physiological methods to measure the human aesthetic response to exact fractal patterns would further elucidate our responses to such timeless patterns.

## Introduction

Whereas exact fractals are built by repeating a pattern at different magnifications, “statistical” fractals introduce randomness into their construction. This disrupts the precise repetition so that only the pattern’s statistical qualities repeat. Consequently, although exact and statistical fractals are both physically complex due to their repeating patterns, the two families of fractals are not visually identical. Because of their prevalence in nature (Mandelbrot, 1982), behavioral studies have predominantly focused on the human response to statistical fractals (Sprott, 1993; Aks and Sprott, 1996; Spehar et al., 2003, 2015; Hagerhall et al., 2004; Taylor et al., 2005, 2011; Forsythe et al., 2011; Spehar and Taylor, 2013).

Sprott (1993) provided the first, systematic investigation of aesthetic responses using fractal patterns generated with equations based on nature’s chaotic processes. He investigated the relationship between aesthetics and fractal dimension, *D*. This parameter quantifies the relative contributions of the coarse and fine scale patterns in the fractal mix of repeating patterns. For images, *D* typically lies in the range 1 < *D* < 2, with a value closer to 2 indicating a larger ratio of fine to coarse scale patterns (Fairbanks and Taylor, 2011). Sprott found that evaluations judged as visually appealing were most often given to the fractal patterns with moderately low fractal dimension (Taylor and Sprott, 2008).

Taylor et al. (1999, 2007) showed that Jackson Pollock’s abstract paintings are composed of statistical fractals, allowing preference studies to be extended to artistic stimuli. Preference was found to peak in the moderately low dimension range for Pollock’s fractals, along with mathematical fractals and for simple fractal objects found in nature (Spehar et al., 2003), a result that has been observed in physiological recordings as well (Taylor, 2006; Taylor et al., 2011). This preference for moderately low dimension was then shown to generalize to the horizon line of complex natural scenes (Hagerhall et al., 2004) as well as gray scale and color images (Taylor et al., 2005; Forsythe et al., 2011; Spehar and Taylor, 2013; Spehar et al., 2015). Abstract, graphical and representational art share these scale-invariant properties (Mureika et al., 2004; Graham and Field, 2007, 2008; Redies et al., 2007; Graham and Redies, 2010). This mounting evidence leads to the conclusion that there is a “universal” preference for statistical fractals that have moderately low dimensionality, but the question of whether this extends to exact fractals remains unanswered.

To date, only Hagerhall et al. (2015) have performed controlled experiments on exact fractals. Their results provide an intriguing hint that non-random, recursive patterns may have a different aesthetic than their statistical counterparts. By taking continuous electrophysiological recordings from the scalp using electroencephalography (EEG), Hagerhall et al. (2015) showed that alpha-band power of the EEG signal changes as a statistical fractal is morphed into its exact counterpart. Here, we report the first systematic study of behavioral responses to exact fractal patterns.

The fractal dimension of an exact fractal is quantified by the expression *D* = log(*N^R^*)/log(1/*S^R^*), where *N* is the number of line segments in the pattern, *S* is the scale factor, and *R* is the number of recursions of the pattern. The number of recursions therefore changes the observed pattern, but not its fractal dimension, and an infinite number of patterns could be generated which have the same dimension (Mandelbrot, 1977). Here, we manipulate these fractal parameters to identify commonalities in response patterns to multiple fractal patterns. Specifically, we tune *D* by varying the relative scaling at each recursion. We consider various generator patterns to investigate the impact of spatial symmetries, such as radial and mirror symmetry, at the same levels of *D* across the patterns. We also vary the level of recursion, the number of repetitions across scales, which we call scale-invariance. Exact scale-invariance has not been investigated previously with regard to aesthetics. We find it worthy of consideration because many aesthetically pleasing patterns that humans generate are strictly ordered and exhibit structure across multiple scales of measurement. For example, Escher’s *Circle Limit* series possesses mirror, radial and scaling symmetries (van Dusen and Taylor, 2013), and represents a recent example of the spatial symmetry and scale-invariance that are held sacred in so many cultures’ art from antiquity onward.

Fractals’ scale invariance represents an intriguing geometry in terms of aesthetics. Fractals possess two features that historically have attracted much interest—complexity and order. Birkhoff (1933) first formalized aesthetic value as the ratio of order and complexity. Complexity, to Birkhoff (1931, 1933), was a physical, measurable characteristic that could be described mathematically, as opposed to the psychological construct that has more recently been introduced by various authors (e.g., Attneave, 1954). We disambiguate the two by calling the latter “perceived complexity” as opposed to Birkhoff’s (1931) complexity, which we label “physical complexity.” Physical complexity was described by Birkhoff (1933) as a physical property of a stimulus that requires automatic adjustments in attention, specifically the number of indefinitely extended lines that cover all of the segments of a polygon, for visually presented stimuli. Birkhoff (1933) described order and symmetry as synonymous, with order operationalized as a linear sum of values that code for the presence of symmetry, vertical mirror symmetry, rotational symmetry, and an ability to be translated for tessellation, minus a value that codes for irregularity in form. This served as the first mathematical description of aesthetic value: the order terms divided by physical complexity (Birkhoff, 1931). Birkhoff’s relationship has failed to predict the response trends in various studies (Eysenck, 1941, 1942; Munsinger and Kessen, 1964a,b; Boselie and Leeuwenburg, 1985; Martindale et al., 1988). As such, others have refined the relationship. Eysenck (1941) was the first to do this, proposing that this relationship is instead multiplicative, with aesthetic value being driven upward by an increase in order or complexity.

Physical complexity, as Birkhoff (1933) notes, is interesting at the more basic level of attention and perceptual processing as well, not just as an aesthetic concept. Attneave (1957) empirically determined that judgments about perceived complexity are dependent upon two of the three factors that he varied—the number of line segments that composed a polygon and whether the pattern was mirror symmetric (whether the edges were curved or straight did not contribute to perceived complexity judgments). Berlyne (1971) extended this in the direction of our study by considering patterns, not just polygons, as a means to determine the factors that affect interestingness and pleasantness. He attempted to generate a more exhaustive list of ways that patterns may vary. Of the two factors Berlyne concluded impact such judgments, his discussion of physical complexity (referred to by Berlyne simply as complexity) is of interest here. His definition of physical complexity consisted of several pattern properties that may be classified as affecting the numerosity of the pattern contents (e.g., amount of material, number of independent units) and the symmetry of the pattern (e.g., asymmetry, random redistribution, irregularity of shape). Thus, across several decades of work, an inconsistent set of definitions for “complexity” has emerged. What is evident, having been consistently observed, is that symmetry and numerosity of the contents of a stimulus composed of Euclidian shapes (i.e., aspects of physical complexity) affects judgments of perceived complexity (Cutting and Garvin, 1987; Eysenck, 1941, 1942).

With the introduction of fractal geometry by Mandelbrot (1977) and the notion that physical complexity is affected by the introduction of structure at increasingly fine scales came the possibility of disrupting the implicit notion that patterns which are not mirror or radially symmetric lack order. Cutting and Garvin (1987) introduced scale-invariance to the study of perceived complexity judgments by presenting participants with images of exact fractals that varied in *D*, the level of recursion, and the number of segments in the generator, and found that these correlate with traditional measures of physical complexity such as the perimeter-area ratio, number of segments, and structural codes (as in Boselie and Leeuwenburg, 1985), thereby generalizing the perceived complexity literature to fractals. Cutting and Garvin (1987) did not find that symmetry affected perceived complexity judgments for their fractal patterns, but this was due to the fact that they only used radially symmetric exact fractals. A major limitation of their study as it relates to this discrepancy is that their stimulus set only included exact fractals. The distinction between exact and statistical fractals is important here in that Berlyne’s (1971) discussion of the preference for physical complexity includes randomization as a factor that contributes to irregularity, with more irregularity being less pleasing. In terms of Berlyne’s (1971) dichotomy of more or less irregular forms, Cutting and Garvin (1987) only used the less-irregular type, exact fractals, and so it has remained unknown whether perceived complexity or preference is affected by order (symmetry) in fractal patterns.

Given that symmetric geometric patterns feature prominently in many cultures’ traditional and contemporary art (Voss, 1998; Graham and Redies, 2010; Koch et al., 2010; Melmer et al., 2013) and that symmetry is detected by the primate visual system (Sasaki et al., 2005) and affects brain responses during aesthetic judgments (Jacobsen et al., 2006), we hypothesized the following: (1) across families of exact fractals, there should be a universal pattern of appeal that varies with physical complexity (more complexity, as quantified by a higher *D* value, should be more preferred); and (2) the visual appeal across *D* should be modulated by more typically studied forms of symmetry (i.e., mirror and radial symmetry) and the level of recursion in the pattern (a greater number of iterations and more symmetry should be more preferred than lower numbers of iterations or less spatial symmetry, thus modulating the appeal of higher *D* patterns).

From these aesthetics and perceived complexity studies, there are two patterns of results describing the change in preference across *D* that may reasonably be expected: a linearly increasing relationship or a quadratic trend that peaks at moderately low *D*. The first is supported by a combination of theory and evidence. There is a strong, positive correlation between judgments of perceived complexity and *D* for exact fractals (Cutting and Garvin, 1987). Theories from Birkhoff (1933) onward predict that physical complexity has a direct effect on aesthetics. If the physical complexity-preference relationship holds for exact fractals and follows from the relationship between *D* and perceived complexity, then we would expect a strong positive linear relationship between preference ratings and *D*.

This contrasts with the prediction that follows directly from the large body of literature that shows that preference universally peaks at moderately low *D* in aesthetic studies of statistical fractals (Spehar et al., 2003, 2015; Hagerhall et al., 2004; Taylor et al., 2005; Forsythe et al., 2011; Spehar and Taylor, 2013). If there is no sensitivity to spatial symmetry as suggested by the Cutting and Garvin (1987) results, spatially asymmetric and spatially symmetric exact fractals should follow the preference patterns observed in those studies of statistical fractals. The Berlyne (1971) aesthetics model predicts a peak as a function of familiarity and physical complexity, but we remain agnostic about where such a peak would occur because there have been no studies of the prevalence of exact fractals of various fractal dimensions. Moreover, evidence of such a peak has not always been observed (Martindale et al., 1990; Nadal et al., 2010; Vessel and Rubin, 2010; Forsythe et al., 2011). Here, we show that the universal aesthetic response to statistical fractals does not generalize to exact fractal patterns with spatial and scale-invariance.

## Experiment 1—Preference for Exact Midpoint Displacement Fractals

### Introduction

We first explored the role of physical complexity in determining preference for an exact fractal pattern by varying the fractal dimension. We held the variables recursion and symmetry constant for our first study, because no previous studies have investigated the aesthetic response to exact fractals, and so we wanted a pure test of the effects of changing *D* of an exact fractal pattern.

### Methods

#### Stimuli—Exact Midpoint Displacement Fractals

**Figure 1:**
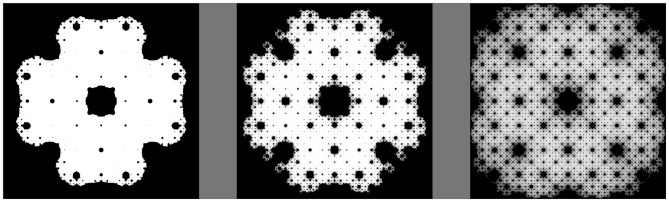
**Exact midpoint displacement fractals with 10 levels of recursion at dimension (A) 1.1, (B) 1.5, and (C) 1.9**.

Exact midpoint displacement fractals (examples shown in Figure 1 and described in Table 1) were generated according to the algorithm described by Fournier et al. (1982). This process starts with four points. As a zero-order level of recursion, a vertex is added to the middle of the square, dividing it into four quadrants. The vertex, *V*, is displaced vertically by a value that is scaled by a factor of 1/2 raised to a power of −2(3−*D*)(*R* + 1), where *D* is fractal dimension and *R* is the current level of recursion. For these exact fractals, the scaling value is held constant for each vertex at each level of recursion, such that *V* = 1*2^−2(3−*D*)(*R* + 1)^. The midpoint of each quadrant then forms a vertex for the first level of recursion that is displaced by *V*. This is iterated for the desired level of recursion, which is 10× in the present study. This allowed us to produce nine 1025 × 1025 pixel images, which can be thought of as terrains that occupy more than a 2-D but less than a 3-D Euclidian space. To achieve a constant level of luminance, we converted these terrains into binary images by applying a threshold at the median. Pixels with lower values were set to zero (black), and values higher than the median set to one (white) as can be seen in Figure 1.

**Table 1:** **Fractals’ characteristics**.

Fractal name	Fractal generator	Dimension range	Mirror symmetry	Radial symmetry	Recursions	Generator segments
Midpoint displacement	Raise midpoint of square	1.1–1.9	+	+	10	4
Sierpinski carpet	Remove square	1.1–1.9	+	+	4	9
Symmetric dragon	Split line segment at midpoint	1.1–2.0	−	+	10	2
Golden dragon-10	Split line segment off midpoint	1.1–1.9	−	−	10	2
Golden dragon-17	Split line segment off midpoint	1.1–1.9	−	−	17	2
Koch snowflake-5	Raise middle third of line segment	1.1–2.0	+	+	5	12
Koch snowflake-6	Raise middle third of line segment	1.1–1.9	+	+	6	12

#### Participants

Forty-two (26 female) undergraduates participated for course credit. Participants’ ages ranged from 18 to 49 (*M* = 20, *Median* = 19). One participant did not report his age.

#### Procedure

This study was carried out in accordance with a protocol approved by the Research Compliance Services of the University of Oregon. All participants gave written informed consent in accordance with the Declaration of Helsinki. Participants were tested individually on Dell Optiplex 755 computers with 1024 × 768 resolution and 60 Hz refresh rate. All participants completed the preference task and then a survey that included items about demographic and personality variables.

At the start of the preference task, participants were instructed to rate the visual appeal of each image using a 7-point scale, where 1 was very low and 7 was very high. There were three practice trials followed by 90 experimental trials. The three practice trials sampled the range of dimension, such that *D* = 1.1, 1.5 and 1.9, while the experimental trials sampled the range of dimension at intervals of 0.1, such that *D* = 1.1, 1.2, …, 1.9. Image presentation order was randomized for each participant on the practice and experimental trials.

All stimuli were presented on a gray background with illumination halfway between the black and white portions of the stimulus. The start of each trial was indicated by a red fixation point, which remained on the screen for one second. Then participants were presented with a fractal stimulus for 3 s. Once, the stimulus was removed, participants indicated visual appeal by pressing the corresponding key on the keyboard. Participants had 3 s to respond before automatically moving to the next trial. If the participant responded before 3 s had passed, the next trial would begin immediately following their response.

All participants saw images with nine different levels of dimension 10×. The images were presented in random order without replacement.

### Results

#### Preference for Exact Midpoint Displacement Fractals Across Dimension

**Figure 2:**
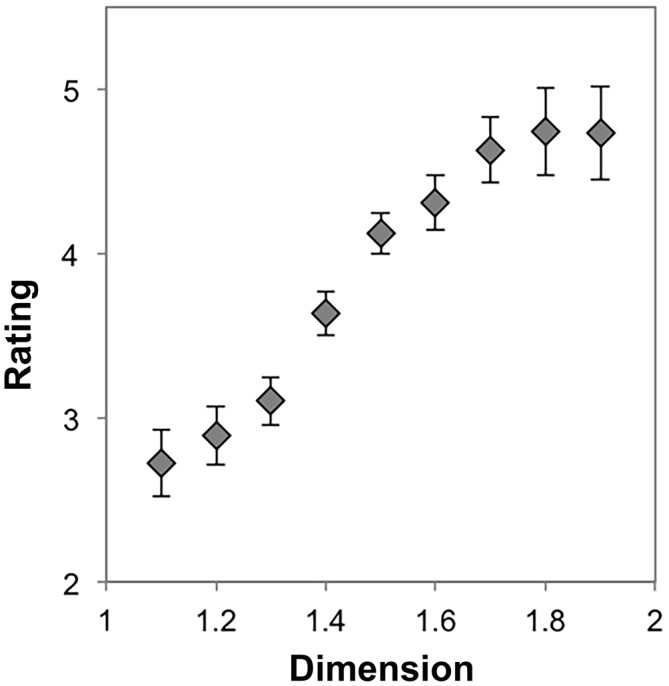
**Mean preference ratings for exact midpoint displacement fractals as a function of dimension (error bars represent standard error)**.

To determine the effect of dimension on preference for exact fractals, we performed a repeated measures ANOVA with nine levels (*D* = 1.1, 1.2, …, 1.9) using each participant’s average preference rating for each stimulus. Mauchly’s test indicated that the assumption of sphericity had been violated, χ^2^_(35)_ = 574.46, *p* < 0.001. Therefore degrees of freedom were corrected using Greenhouse-Geissser estimates of sphericity (*ε* = 0.16). The results show that there was a significant effect of dimension on preference, *F*_(1.30,51.92)_ = 21.71, *p* < 0.001, η^2^ = 0.35. Within-subject contrasts showed significant linear (*p* < 0.001, η^2^ = 0.38), quadratic (*p* < 0.007, η^2^ = 0.17), cubic (*p* < 0.001, η^2^ = 0.34), 6th (*p* = 0.02, η^2^ = 0.14), and 7th (*p* = 0.03, η^2^ = 0.12) order trends. Other higher-order trends were non-significant (*p* > 0.05 and η^2^ < 0.10 for all other trends). The trend is predominantly linear and cubic in Figure 2, suggesting that preference increases with *D* for exact fractals and stabilizes at high *D*.

#### Subgroup Preferences for Exact Midpoint Displacement Fractals Across D

To determine whether the observed trend could be better explained as a combination of multiple discrete subgroups’ patterns of responses, we performed a two-step cluster analysis and tested for an interaction between the subgroups’ preferences and *D*. First, we performed a hierarchical cluster analysis using Ward’s method to separate individuals into groups using their preference ratings for each level of *D*. The resultant agglomeration matrix indicated a two-cluster solution. We then performed *k*-means clustering analysis to form two groups. These cluster analysis techniques are described in more detail in Norušis (2011).

**Figure 3:**
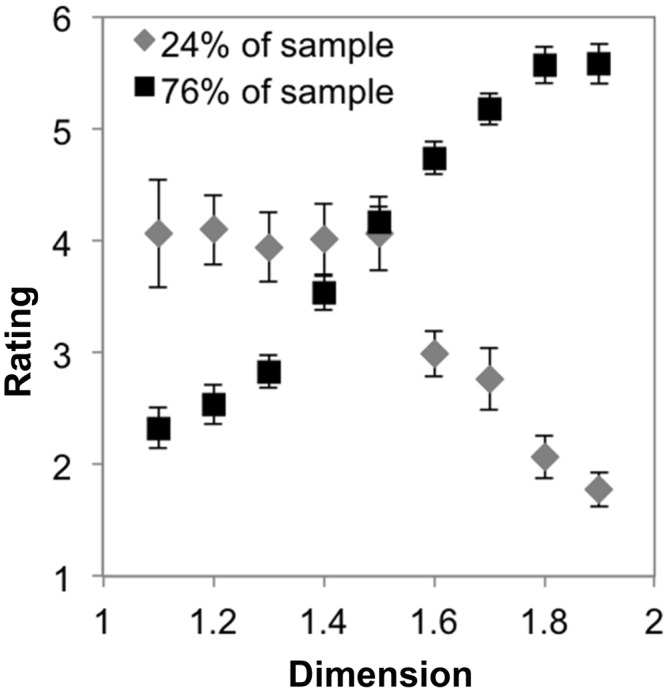
**Mean preference ratings for exact midpoint displacement fractals as a function of dimension for each subpopulation identified with cluster analysis (error bars represent standard error)**.

Finally, we investigated whether there was an interaction between cluster-membership and *D* by performing a mixed ANOVA with nine levels of dimension and two groups. Mauchly’s test indicated that the assumption of sphericity had been violated, *χ*^2^_(35)_ = 270.02, *p* < 0.001. Therefore degrees of freedom were corrected using Greenhouse-Geissser estimates of sphericity (*ε* = 0.28). The results show that there was a significant interaction between *D* and group, *F*_(2.52,87.81)_ = 79.81, *p* < 0.001, η^2^ = 0.67. Figure 3 shows that a minority of subjects (24%, gray line) prefer lower *D* midpoint fractals, with preference ratings decreasing as *D* increases, on average. In contrast, preference ratings from the majority of subjects (76%) increase as a function of *D* (Figure 3, black line). This refines our interpretation about the sample’s average preferences.

We do not report the between-subjects test because, while significant, it is not theoretically interesting given that the clusters were formed through the identification of differences between the groups. We also leave out the main effect of dimension for this test, like the main effects of and interactions among within-subject variables for these group-wise tests because if they remain significant, they have been reported above, whereas if they do not, it may be that they have lost significance due to the loss of statistical power.

### Discussion

Our results suggest that there is typicality of preference for a particular level or range of levels of exact fractal patterns’ fractal dimension. For most individuals, the most preferable patterns are of the highest *D*, and the least preferable are of the lowest *D*, as shown in Figure 2. Because a minority of individuals prefer lower *D* midpoint fractals (see Figure 3), it is possible that there are individual differences to be explored in future studies.

The pattern of typical preferences for exact fractals differs from that of preference for statistical fractals. Multiple studies have shown that preference for statistical fractals peaks at low-to-moderate levels of *D* (Sprott, 1993; Aks and Sprott, 1996; Spehar et al., 2003; Taylor et al., 2005; Spehar and Taylor, 2013), whereas peak preference for exact fractals appears to trend toward a value near *D* = 2 for most people in this experiment. A particularly acute limitation of this study is that we only presented participants with one fractal pattern at a particular level of recursion. Our result might not generalize to patterns where the level of recursion and extent of spatial symmetry is different, a possibility we test in Experiment 2.

## Experiment 2—Preference for Exact Fractals Across Dimension, Recursion and Symmetry

### Introduction

After finding that the pattern of typical preference for exact midpoint displacement fractals is distinct from the pattern of preferences that has been observed for statistical fractals, we were intent on testing the generalizability of our first study’s results. Here, we manipulate the variables recursion and spatial symmetry by presenting participants with six fractal patterns that each vary in fractal dimension as in Experiment 1.

### Methods

#### Stimuli

**Figure 4:**
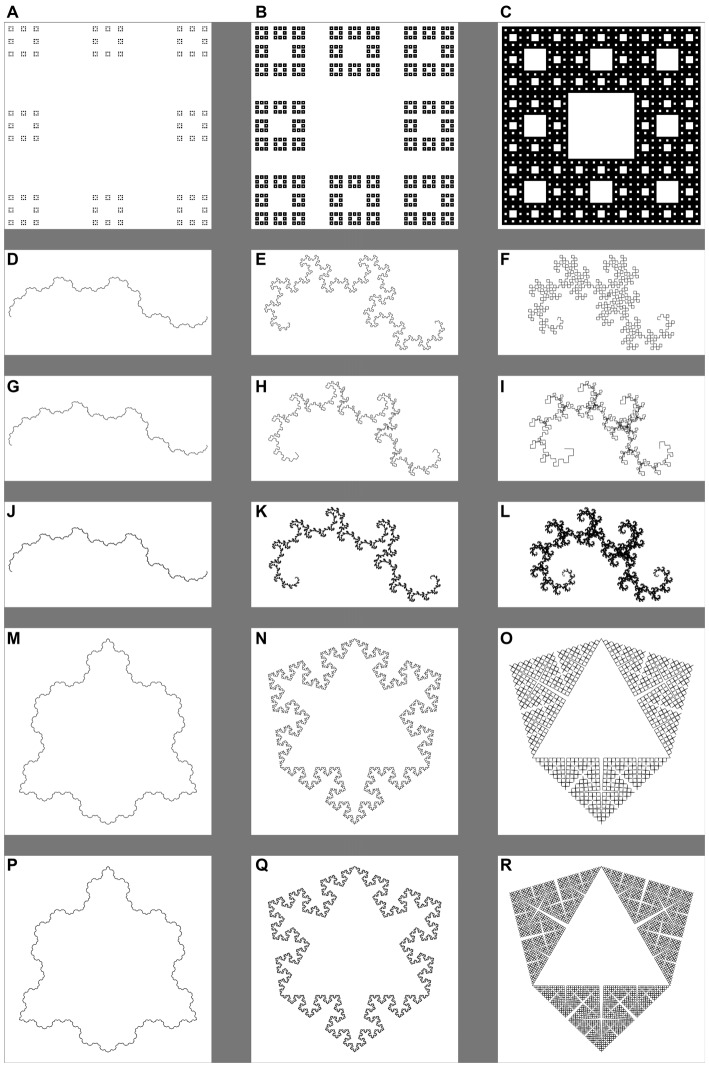
**Exact fractals that vary in generator rule, level of recursion and dimension.** Each column shows a different level of dimension, such that the dimension of the fractals in columns 1 **(A,D,G,J,M,P)** 2 **(B,E,H,K,N,Q)** and 3 **(C,F,I,L,O,R)** is 1.1, 1.5, and 1.9, respectively. **(A–C)** Show Sierpinski carpets, where the middle 1/9th of the pattern has been removed at four scales. **(D–L)** Show dragon fractals that vary in symmetry recursion and dimension. **(D–F)** (Dragons) result from 10 levels of recursion with bisection of the segments at each level of recursion. **(G–I)** (Golden dragons) result from 10 levels of recursion with ratio a:b, where a = (1/φ)^(1/φ)^ and b = a^2^, as the basis of the adjustment of each segment at each level of recursion. **(J–L)** (Golden dragons) result from 17 levels of recursion with the same rule as **(G–I)**. **(M-R)** Show Koch snowflakes that vary in recursion and dimension. **(M–O)** Result from bisection of the segments of a triangle and bisection of each segment at each level of recursion for five recursions. **(P–R)** Are the result of an additional level of recursion (six recursions) applied to the snowflakes of **(M–O)**.

For this study, we generated images of several types of fractals for 1 to 20 recursions at multiple *D* values, much like Cutting and Garvin (1987). From those, we chose four fractal generator rules and tested one or two levels of recursion for each, resulting in six patterns that varied in *D*, recursion, and spatial symmetry, which we presented to participants. Specifically, we presented participants with one set of Sierpinski carpets with four levels of recursion, symmetric dragon fractals with 10 levels of recursion, two sets of asymmetric dragon fractals—one with 10 and another with 17 levels of recursion, and two sets of Koch snowflake fractals—one with four and the other with five levels of recursion. Examples from these stimulus sets are shown in Figure 4, and they are described in Table 1. For examples of the effects of recursion, see Figure panel pairs 4G–J, 4G–J, H–K, I–L, M–P, N–Q, and O–R. For a further description of these patterns’ generation method and visualization of the process of recursion, see Barnsley et al. (1988) or Cutting and Garvin (1987).

##### Sierpinski Carpet Fractals

To generate Sierpinski carpet fractals, we start with a filled area, for example from [0, 0] and [0, 1] to [1, 0] and [1, 1]. For the zero-order recursion, we remove a portion, such as the middle ninth, from [0.33, 0.33] and [0.33, 0.67] to [0.67, 0.33] and [0.67, 0.67]. This process is iterated for each region (each 1/9th) of every section that was not removed at the previous level recursion for a specified number of recursions. We utilized images of Sierpinski carpets that had undergone four levels of recursion. These fractal patterns exhibit the spatial symmetry of the exact midpoint displacement fractals used in Experiment 1.

##### Symmetric Dragon Fractals

To generate dragon fractals, we start with a line segment that extends from [0, 0] to [1, 0]. For the zero-order recursion, we break the segment into two parts by raising a point between these two by a particular value (e.g., [0.5, 0] may become [0.5, 0.5]) such that this new pair of line segments and the original segment would form a triangle. The original segment is removed and the process is iterated for each new line segment at each level of recursion for a specified number of recursions. We manipulated the scaling dimension of these fractals by adjusting the angle at which the new segments are joined at each recursion. We utilized images of dragon fractals that had undergone 10 levels of recursion. These fractal patterns exhibit only radial and not mirror symmetry.

##### Golden Dragon Fractals

We call these golden dragons because the line segments are generated using φ (although their lengths do not scale at 1:1.6). The lengths of the segments that replace the previous recursion level’s segment are given by the equations a = (1/φ)^(1/φ)^, b = [(1/φ)^(1/φ)^]^2^, and c^2^ = a^2^ + b^2^, such that a ≠ b, whereas a = b in the symmetric dragon fractals. We manipulated the scaling dimension of these fractals by adjusting the angle at which the new segments are joined at each recursion as in the symmetric dragon fractals.

We chose recursion levels of 10 and 17 to survey the range of recursions and differentiate between moderate and high levels of recursion for a pattern with a base of one segment, and to provide a pattern comparable to the symmetric dragons of this experiment and the midpoint displacement fractals of Experiment 1.

These fractal patterns do not exhibit spatial symmetry, as the radial symmetry, which was exhibited by the other dragon fractals, is disrupted by our use of the golden ratio to define the two sub-units of each level of recursion.

##### Koch Snowflake Fractals

To generate Koch curves, the same starting line segment is used, but only a portion of the segment is raised. At the first level of recursion, that rule is carried out on each of the sub-sections, such that four midsections are then raised on the next recursion where a single segment existed prior to the previous recursion.

We tessellated the final pattern twice to form a snowflake pattern with multiple axes of reflection and radial symmetry. Because the edge segments grow at a much more rapid rate (twice as fast as the dragons), and because we tessellated the pattern, we chose *a priori* to use the Koch snowflake images that showed five and six levels of recursion to more closely match the visual appeal of the asymmetric dragons which have greater recursion.

These fractal patterns exhibit radial and mirror symmetry, with a different number of axes of radial and mirror symmetry than the exact midpoint displacement fractals used in Experiment 1 or the Sierpinski carpets used in this Experiment.

#### Participants

Eighteen undergraduates (7 female) participated for course credit. Participants’ ages ranged from 18 to 24 (*M* = 20, *Mdn* = 19).

#### Procedure

This study was carried out in accordance with a protocol approved by the Research Compliance Services of the University of Oregon. All participants gave written informed consent in accordance with the Declaration of Helsinki. Participants were tested individually on Dell Optiplex 755 computers with 1024 × 768 resolution and 60 Hz refresh rate. All participants completed the preference task described in Experiment 1 using the stimuli described in “Introduction” Section and its subsections.

Each trial consisted of the presentation and opportunity to rate one fractal pattern at a given level of dimension, recursion, and symmetry. Each trial began with the presentation of a fractal pattern that remained on the screen for 3 s. Once the stimulus was removed, participants indicated visual appeal by pressing a numeric key in the range of 1 to 7. Participants had 3 s to respond before automatically moving to the next trial. If the participant responded before 3 s had passed, the next trial would begin immediately following their response. Trials were presented within blocks.

Blocks consisted of only one fractal generator and recursion level to mitigate cross-pattern comparisons. Each block began with three practice trials. The practice trials consisted of the presentation of an image at *D* = 1.1, 1.5, or 1.9, and a subsequent fixation period during which the participant was asked to respond. During each block’s experimental trial period, an image from each dimension level was presented once. There were nine experimental trials per block. Practice and experimental trial stimulus order was randomized without replacement.

Block order was randomized without replacement. The block sequence was repeated three times with new random orders for each repetition for each participant. Participants completed a total of 18 blocks.

Participants’ responses to each stimulus were averaged to give a continuous preference rating that could span the range 1 to 7.

Two stimuli (a 5-recursion Koch snowflake and a 10-recursion symmetric dragon), each with *D* = 2.0, were presented to participants within their respective pattern-recursion blocks, but have been left out of the analyses that follow. They are not reported for theoretical reasons—here we are only interested in fractals that would not fill space at an infinite number of recursions. We are not interested in the aesthetics of shapes that can be described by Euclidian geometry (e.g., a Sierpinski carpet with *D* = 2.0 would be a black square).

### Results

We performed three analyses, one on each of the sets of fractals that we only tested at one level of recursion, and another on the sets of fractals that we tested with two levels of recursion. This allowed us to: (1) generalize our findings from the previous experiment’s exact fractals to several different sets of exact fractals that exhibit radial and mirror symmetry; (2) test whether the results from Experiment 1 generalize to line fractals that do not exhibit the extensively studied property of mirror symmetry; and (3) test for interactions among dimension, recursion and spatial symmetry.

#### Preference for Sierpinski Carpets—Another Fractal with Radial and Mirror Symmetry

**Figure 5:**
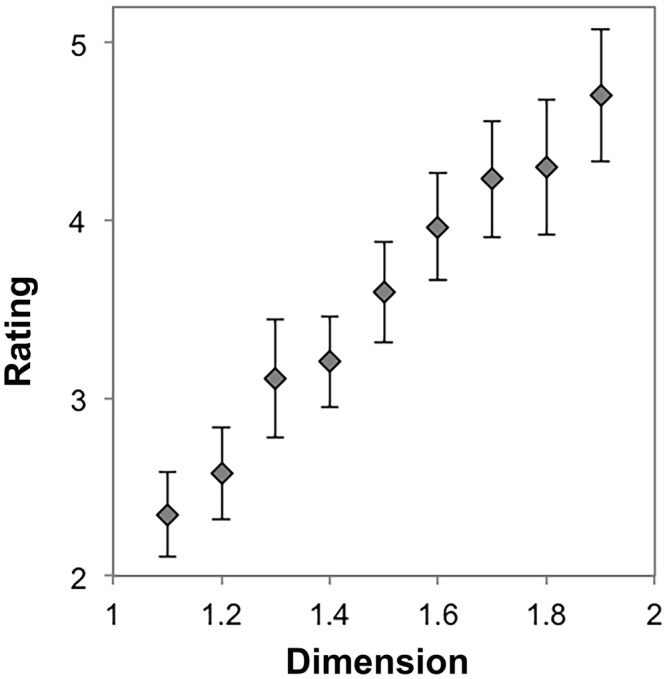
**Mean preference ratings for Sierpinski carpet fractals as a function of dimension (error bars represent standard error)**.

To determine whether the trend we observed in our first experiment generalizes to other exact fractals, we performed a repeated measures ANOVA with nine levels (*D* = 1.1, 1.2, …, 1.9) using each participant’s average preference rating for each stimulus from a set of Sierpinski carpets. Mauchly’s test indicated that the assumption of sphericity had been violated, *χ*^2^_(35)_ = 91.21, *p* < 0.001. Therefore degrees of freedom were corrected using Greenhouse-Geissser estimates of sphericity (*ε* = 0.28). The results show that there was a significant effect of dimension on preference, *F*_(2.25,38.30)_ = 18.77, *p* < 0.001, η^2^ = 0.53. Within-subject contrasts showed a significant linear trend (*p* < 0.001, η^2^ = 0.66). All higher-order trends were non-significant (*p* > 0.05 and η^2^ < 0.10 for all other trends). This result is apparent in Figure 5, which confirms that preference increases with *D* for exact fractals that exhibit radial and mirror symmetry.

#### Subgroup Preferences for Sierpinski Carpet Fractals Across D

**Figure 6:**
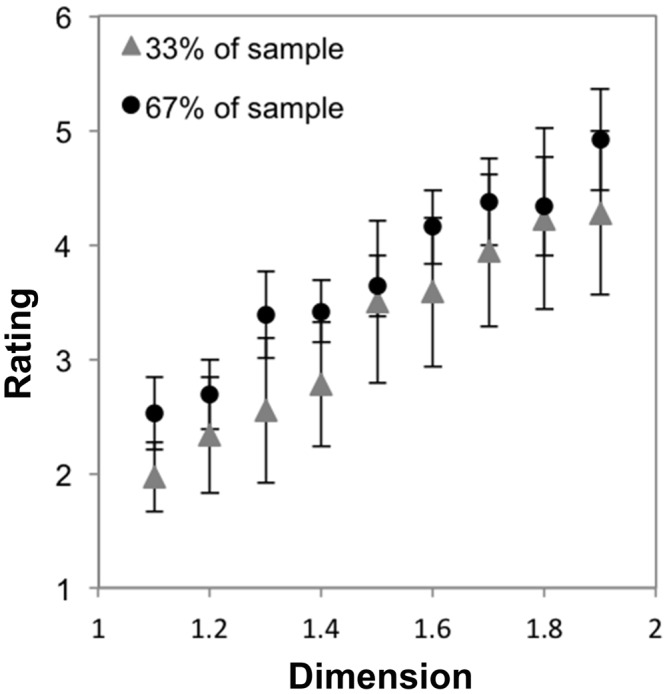
**Mean preference ratings for Sierpinski carpet fractals as a function of dimension for each subpopulation identified with cluster analysis (error bars represent standard error)**.

To test, again, whether there are distinct subgroups who exhibit different preference trends, we performed a two-step cluster analysis as described in “Subgroup Preferences for Exact Midpoint Displacement Fractals across *D*” Section using all of the preference ratings from these participants, which again indicated a two-cluster solution. We tested for an interaction between the subgroups’ preferences and *D* by performing a mixed ANOVA with nine levels of dimension and two groups. Mauchly’s test indicated that the assumption of sphericity had been violated, *χ*^2^_(35)_ = 87.03, *p* < 0.001. Therefore degrees of freedom were corrected using Greenhouse-Geissser estimates of sphericity (*ε* = 0.28). The results show that there was not a significant interaction between *D* and group, *F*_(2.20,35.21)_ = 9.94, *p* = 0.73, η^2^ = 0.02. Figure 6 shows that both groups identified by the cluster analysis tend to indicate increasing preferences across the range of *D*, having used a similar portion of the scale for this subset of stimuli. This strengthens our interpretation about the sample’s average preferences. This suggests that there is a typical pattern of preference for exact fractal patterns that are distributed across the entire image space (here our smaller sample may have failed to include individuals from the subpopulation which prefers lower *D* fractals). The following analyses test whether this extends to line fractals that are not equally distributed across an image space.

#### Preference for Symmetric Dragon Fractals—Line Fractals with Radial Symmetry

**Figure 7:**
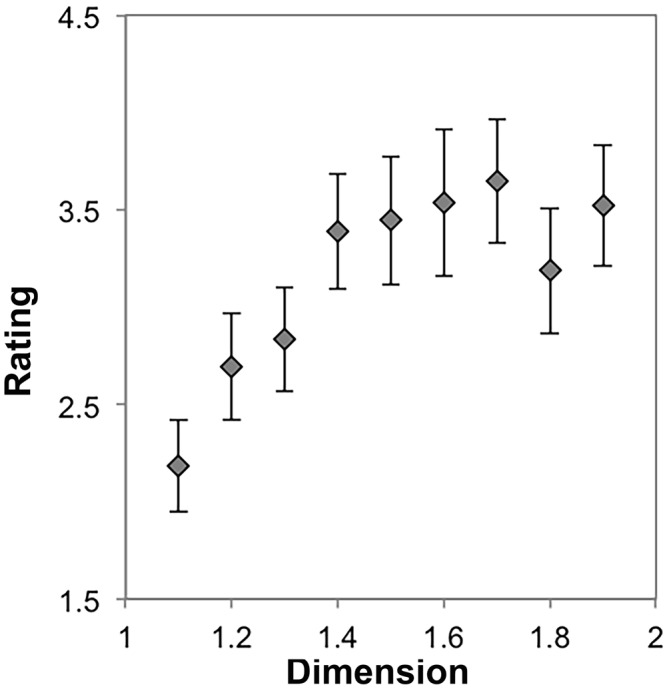
**Mean preference ratings for symmetric dragon fractals as a function of dimension (error bars represent standard error)**.

Because many previous studies have focused on faces and other mirror-symmetric patterns (Rhodes et al., 1999; Thornhill and Gangestad, 1999; Jacobsen and Höfel, 2003; Jacobsen et al., 2006), we wanted to determine whether the trend we observed in our first experiment and the preceding analysis was due, specifically, to an interplay between mirror symmetry and scale-invaraince for patterns that are equally distributed across an image space. To test this, we again performed a repeated measures ANOVA with nine levels (*D* = 1.1, 1.2, …, 1.9) using each participant’s average preference rating for each stimulus from a set of dragon fractals, which exhibit no mirror symmetry, but retain the radial symmetry observable in the stimuli that contributed to the results discussed so far. Mauchly’s test indicated that the assumption of sphericity had been violated, *χ*^2^_(35)_ = 120.66, *p* < 0.001. Therefore degrees of freedom were corrected using Greenhouse-Geissser estimates of sphericity (*ε* = 0.33). The results show that there was a significant effect of *D* on preference, *F*_(2.61,44.39)_ = 6.86, *p* = 0.001, η^2^ = 0.29. Within-subject contrasts showed significant linear (*p* = 0.01, η^2^ = 0.33), quadratic (*p* = 0.009, η^2^ = 0.34), 5th (*p* = 0.02, η^2^ = 0.29) and 7th (*p* < 0.001, η^2^ = 0.53) order trends. All other trends were non-significant (*p* > 0.05 and η^2^ < 0.15 for all other trends). This result is apparent in Figure 7, which shows a more complicated relationship between preference and *D* for exact fractals that do not exhibit mirror symmetry. Here, it appears that preference increases with *D*, to a point, before stabilizing.

#### Subgroup Preferences for Symmetric Dragon Fractals Across D

**Figure 8:**
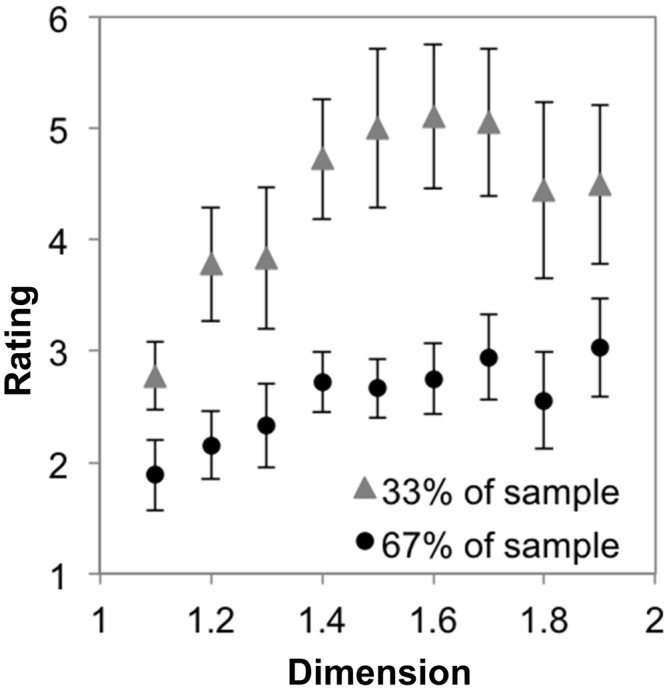
**Mean preference ratings for symmetric dragon fractals as a function of dimension for each subpopulation identified with cluster analysis (error bars represent standard error)**.

To test, again, whether there are distinct subgroups that exhibit different preference trends, we performed a mixed ANOVA with nine levels of dimension and the two groups as described in “Subgroup Preferences for Sierpinski Carpet Fractals Across *D*” Section. Mauchly’s test indicated that the assumption of sphericity had been violated, *χ*^2^_(35)_ = 112.09, *p* < 0.001. Therefore degrees of freedom were corrected using Greenhouse-Geissser estimates of sphericity (*ε* = 0.31). The results show that there was not a significant interaction between *D* and group, *F*_(2.49,39.80)_ = 1.47, *p* = 0.24, η^2^ = 0.08. Figure 8 shows that both groups identified by the cluster analysis tend to indicate increasing preferences across the range of *D*, although they used different ranges of the scale for this subset of stimuli. The subtle difference in trends between groups for this set of stimuli in particular is detected by the quadratic within-subject contrast (*p* = 0.02, η^2^ = 0.28) with all other trends non-significant (*p* > 0.05, η^2^ < 0.15). This result further replicates our finding of a generator-pattern insensitive effect of higher preference for high *D* than low *D* exact fractals. The following analyses further probe the consistency of this trend while also allowing us to test for effects of symmetry and level of recursion.

#### Preference for Line Fractals that Vary in Extent of Symmetry and Recursion

Having confirmed that there is typicality in the exact fractals for which the majority of individuals express their highest and lowest preference, we now move to our second hypothesis—that the visual appeal of scale-invariance at different levels of *D* is modulated by more typically studied forms of symmetry and the level of recursion in a pattern. We tested this by manipulating the level of recursion in two generators that differ in their extent of symmetry. These golden dragons have no mirror or radial symmetry, while the Koch snowflakes exhibit multiple axes of radial and mirror symmetry.

Preference ratings for the golden dragons and Koch snowflakes were subjected to a three-way ANOVA having two levels of spatial symmetry (absent [Golden Dragon], present [Koch]), two levels of recursion (low [Dragon 10 and Koch 5], high [Dragon 17 and Koch 6]), and nine levels of fractal dimension (*D* = [1.1, 1.2, …, 1.9]). Degrees of freedom for each *F*-test are reported with Greenhouse-Geissser correction when assumptions of sphericity have been violated, as determined by *p* < 0.05 for Mauchly’s test.

The analysis yielded a main effect of the number of recursions *F*_(1,17)_ = 94.15, *p* = 0.001, η^2^ = 0.85, such that aesthetic value ratings were higher for fractals with a higher number of recursions (*M* = 4.7, 95% CI = [4.37, 5.03]) than fractals with fewer recursions (*M* = 3.59, 95% CI = [3.25, 3.94]). There was also a main effect of the degree of symmetry *F*_(1,17)_ = 17.92, *p* = 0.001, η^2^ = 0.51, such that preference ratings for dragon fractals (*M* = 3.61, 95% CI = [3.19, 3.03]), which have no spatial symmetry, were lower than preference ratings for Koch snowflakes (*M* = 4.68, 95% CI = [4.28, 5.09]), which have both mirror and radial symmetry. There was also a main effect of *D* *F*_(1.76,29.99)_ = 25.87, *p* < 0.001, η^2^ = 0.60, with significant linear (*p* < 0.001, η^2^ = 0.60) and quadratic (*p* < 0.001, η^2^ = 0.84) trends accounting for the majority of the variance (all higher order trends were non-significant (*p* > 0.05, η^2^ < 0.15).

**Figure 9:**
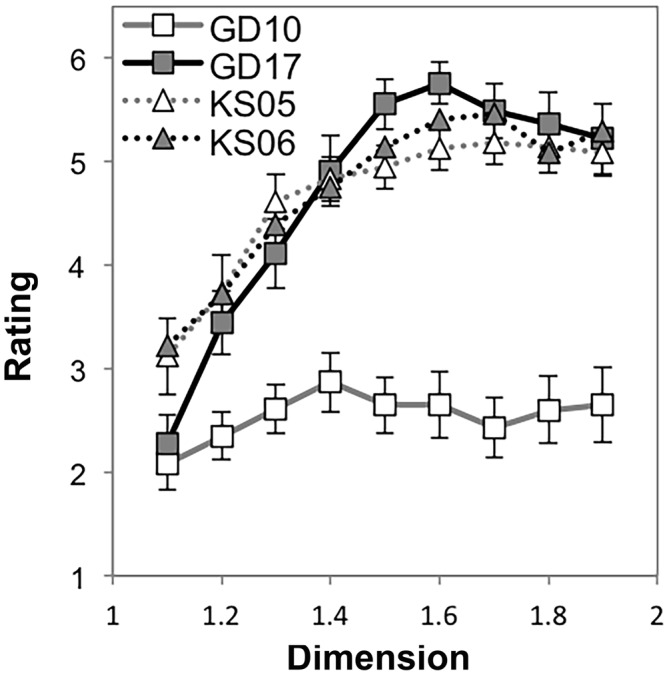
**Mean preference ratings for Koch snowflake and golden dragon fractals as a function of dimension (error bars represent standard error)**.

The analysis also yielded two significant two-way interactions. A significant interaction was observed between symmetry and recursion, *F*_(1,17)_ = 95.57, *p* < 0.001, η^2^ = 0.85. When this is considered in light of the 3-way interaction shown in Figure 9, it is clear that while there is no difference in level of recursion for the Koch snowflakes, the lower-level recursion golden dragon fractal is much less preferable than its higher-level recursion counterpart across the majority of the range of *D* (see Figure 9). There was also a significant interaction between recursion and *D*, *F*_(3.10,52.74)_ = 9.95, *p* < 0.001, η^2^ = 0.37. Again, this is driven by the 3-way interaction and the lower preference for the low-level recursion golden dragon fractal. No significant interaction between level of symmetry and change in *D* was observed *F*_(2.19,37.30)_ = 0.51, *p* = 0.62, η^2^ = 0.03. The two generators appear to follow similar linear-quadratic trends.

These effects are driven by a three-way interaction among dimension, recursion, and symmetry *F*_(3.81,64.82)_ = 11.58, *p* < 0.001, η^2^ = 0.41. The Koch snowflakes with their 3-axis symmetry were rated equivalently across changes in *D* at five and six iterations (see Figure 9). Meanwhile, the golden dragon fractal with 10 recursions was given a largely constant preference rating across *D* that was much lower than the ratings given the 17 recursion dragon fractal at most fractal dimensions. The difference appears to increase as a function of *D* (see Figure 9). These differences are characterized by significant linear (*p* < 0.001, η^2^ = 0.57) and quadratic (*p* < 0.001, η^2^ = 0.55) trends. All higher order trends were non-significant (*p* > 0.05, η^2^ < 0.05).

Although we have characterized the within-participants effects of dimension, recursion and symmetry here, the test also yielded a large, significant between-subjects effect *F*_(1,17)_ = 773.64, *p* < 0.001, η^2^ = 0.98. We do not have sufficient power to investigate individual differences with our sample size, but speculate that this, in part, is due to some participants whose preferences diverge from those of the majority as observed in Experiment 1 and the preceding analyses.

#### Subgroup Preferences for Line Fractals that Vary in Extent of Symmetry and Recursion

**Figure 10:**
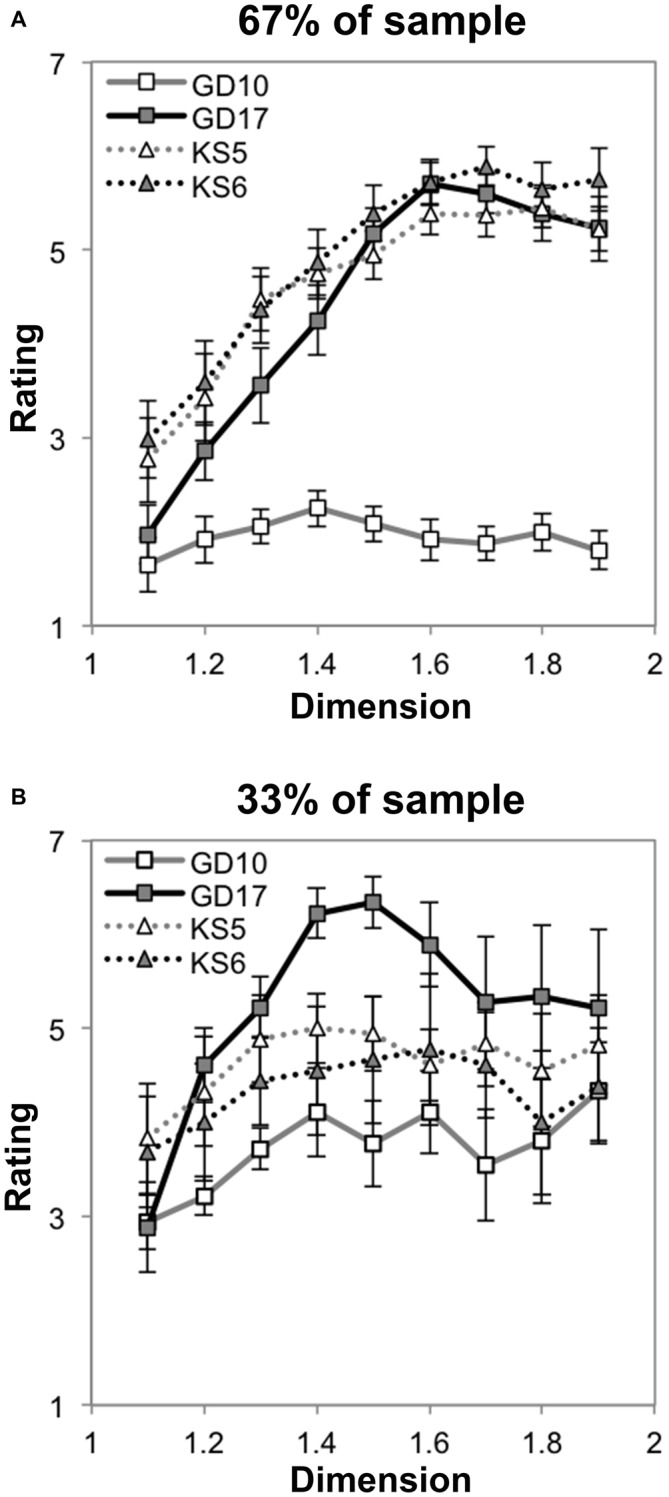
**Mean preference ratings for Koch snowflake and golden dragon fractals as a function of dimension for each subpopulation identified with cluster analysis (error bars represent standard error).** The majority (67%) subpopulation’s preference ratings are shown in **(A)**, while the minority (33%) subpopulation’s preference ratings are shown in **(B)**.

To test whether these trends varied by subgroup, we performed a mixed ANOVA with two levels of symmetry, two levels of recursion, nine levels of dimension and the two subgroups as described in “Subgroup Preferences for Sierpinski Carpet Fractals Across *D*” Section. Mauchly’s test indicated that the assumption of sphericity had been violated for each of the effects involving dimension, so degrees of freedom were corrected using Greenhouse-Geissser correction. At the levels of 2- and 3-way interaction, we only observed a significant interaction between group and symmetry, *F*_(1,16)_ = 21.39, *p* < 0.001, η^2^ = 0.57. This is driven by a four-way interaction among dimension, recursion, symmetry and subgroup *F*_(3.42,54.69)_ = 7.91, *p* < 0.001, η^2^ = 0.33. Figure 10 shows a striking difference between groups in that the majority of individuals express low preference for asymmetric line fractals with low levels of recursion. Interpreted in conjunction with the effect observed in “Subgroup Preferences for Symmetric Dragon Fractals Across *D*” and “Preference for Line Fractals that Vary in Extent of Symmetry and Recursion” Sections, it seems that the majority of individuals prefer high *D* fractals that exhibit symmetry and/or a high number of recursions, while the preference ratings of the minority group appear to saturate at moderate *D*. Still, it is noteworthy that both subgroups maintain the generator-pattern insensitive effect of higher preference for high *D* than low *D* for exact fractals.

#### Effect of Symmetry in Dragon Fractals

Our data also lends itself to tests of the effects of symmetry. First, we consider whether the presence of radial symmetry without mirror symmetry impacts preference for a fractal, and whether this differs across the subgroups identified in this experiment. We compared the aesthetic appeal of 10-recursion golden and symmetric dragon fractals to test the hypothesis that the presence of radial symmetry would be more preferred than its absence and that this effect would be enhanced at higher levels of dimension.

Preference ratings for the golden and radially symmetric dragons were subjected to a mixed ANOVA having two levels of radial symmetry (absent [Golden Dragon], present [Symmetric Dragon]), nine levels of fractal dimension (*D* = [1.1, 1.2, …, 1.9]), and the two subgroups. Degrees of freedom for each *F*-test are reported with Greenhouse-Geissser correction when assumptions of sphericity have been violated, as determined by *p* < 0.05 for Mauchly’s test.

The analysis yielded a main effect of symmetry *F*_(1,17)_ = 23.14, *p* < 0.001, η^2^ = 0.58, such that preference ratings for golden dragon fractals (*M* = 2.54, 95% CI = [2.02, 3.06]), were lower than preference ratings for radially symmetric dragon fractals (*M* = 3.16, 95% CI = [2.63, 3.69]). There was also a main effect of *D, F*_(1.61,27.28)_ = 6.04, *p* = 0.01, η^2^ = 0.26, with a strong quadratic trend (*p* = 0.001, η^2^ = 0.57) and idiosyncratic differences in preference ratings resulting in significant linear, cubic, and 7th order trends as well (*p*s < 0.05); all other trends were non-significant.

**Figure 11:**
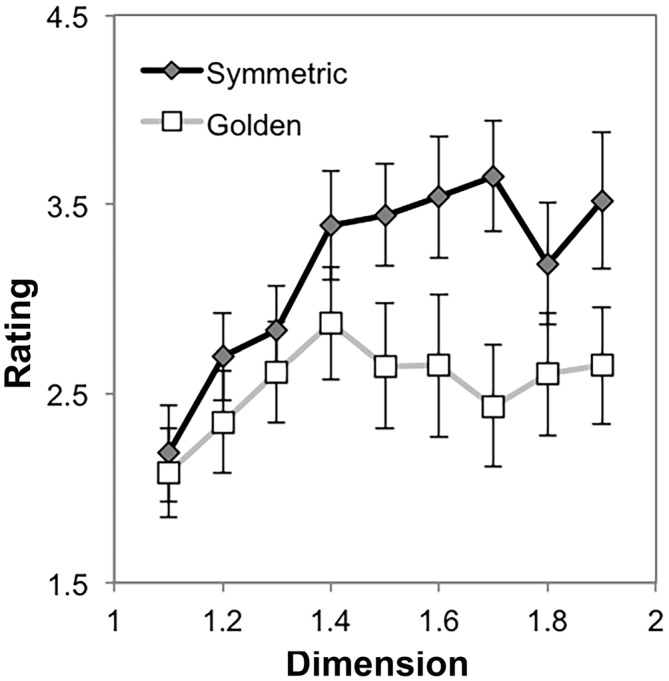
**Mean preference ratings for 10-recursion symmetric and golden dragon fractals as a function of dimension (error bars represent standard error)**.

These main effects should be interpreted in light of a significant interaction between symmetry and *D*, *F*_(3.16,53.76)_ = 2.82, *p* = 0.045, η^2^ = 0.14. Figure 11 shows that as *D* increases, the preference for radially symmetric dragon fractals increases more than preference for golden dragon fractals. This interpretation is consistent with the significant linear trend (*p* = 0.02, η^2^ = 0.28) and likely over-fit but significant 5th and 7th order trends (*p*s < 0.05); all other trends were non-significant.

There were no significant interactions between the within-subjects terms and subgroup factor (all *F* < 1.6, *p* > 0 .05, and η^2^ < 0.10). This indicates that for low levels of recursion, the presence of radial symmetry does not affect the preference ratings of the subgroups differently, which is similar to our findings for Sierpinski carpets and radially symmetric dragon fractals when considered alone.

#### Subgroup Preferences for the Presence of Mirror Symmetry Across Exact Fractals

The preceding analysis suggests that when levels of recursion are low, there is no effect of radial symmetry on preference ratings across the subgroups identified by cluster analysis, but this does not preclude an effect of symmetry at higher levels of recursion or an effect of mirror symmetry. To investigate whether the subgroup preferences differed as a function of mirror symmetry, we formed aggregate scores for fractals lacking mirror symmetry (the average of all preference ratings for dragon fractals) and fractals with mirror symmetry (the average of all preference ratings for Koch snowflakes and Sierpinski carpets) at each level of *D*.

**Figure 12:**
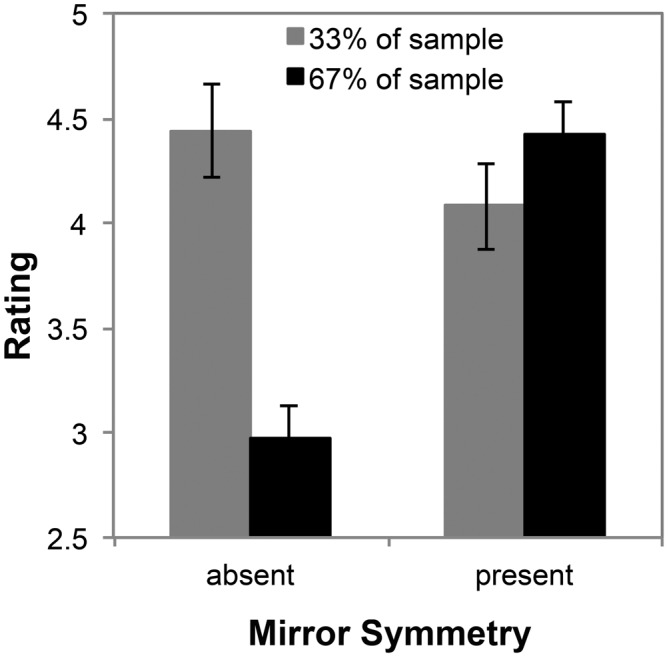
**Mean preference ratings for mirror symmetric (Koch snowflakes and Sierpinski carpets) and non-mirror symmetric (all Dragons) fractals for each subpopulation identified with cluster analysis (error bars represent standard error)**.

Aggregate score for mirror symmetric and non-mirror symmetric fractals at each level of *D* were subjected to a mixed ANOVA with two levels of mirror symmetry and nine levels of *D* for the two subgroups. This revealed a significant interaction between mirror symmetry and subgroup membership, *F*_(1,16)_ = 37.82, *p* < 0.001, η^2^ = 0.70, but not between *D* and subgroup membership *F*_(1.72,27.45)_ = 1.13, *p* = 0.33, η^2^ = 0.07 or among *D*, mirror symmetry and subgroup membership *F*_(1.93,30.92)_ = 1.16, *p* = 0.33, η^2^ = 0.07. The subgroup consisting of the majority of individuals rated mirror-symmetric fractals much higher than the non-mirror symmetric fractals, whereas the minority subgroup’s ratings did not differ with the presence or absence of mirror symmetry, as shown in Figure 12. This result is clearly driven by the low preference ratings given to low-recursion dragon fractals by individuals in the majority subgroup as shown in Figures 8, 10.

### Discussion

In this study, all of our participants showed a consistent preference for higher levels of *D*, affected in subtle ways by the simplicity of the generator pattern, and the extent of symmetry and recursion. Meanwhile, we observed a stark difference in the subpopulation’s responses to the presence or absence of mirror symmetry. This is an interesting and novel finding, given that previous research indicates that people prefer symmetric images (Jacobsen and Höfel, 2003; Cárdenas and Harris, 2006; Jacobsen et al., 2006). We showed that for a minority of individuals, the presence or absence of mirror symmetry does not strongly modulate preference ratings. Still, for most, a lack of mirror and radial symmetry can be overcome by including more recursion and higher fractal dimensionality. Our results underline the importance of symmetry on preference, generally, but reveal that the number of recursions can interact with symmetry in modulating preference across *D*.

## General Discussion

Across these two studies we found that preference is higher for higher *D* fractal patterns when they are ordered, in contrast to studies that have found that low to moderate *D* fractals are most preferred when the patterns are statistical (Sprott, 1993; Aks and Sprott, 1996; Spehar et al., 2003; Hagerhall et al., 2004, 2015; Taylor et al., 2005, 2011; Spehar and Taylor, 2013). The consistency of this effect across various exact fractals lends itself to the conclusion that there is universality of preference for higher fractal complexity for exact fractals that contrasts with the universality of preference for lower *D* statistical fractals that Sprott (1993) first began to detect.

Typicality in preference for exact fractals allows us to extend the predictions and theory that relate physical complexity and aesthetics from Birkhoff onward. Consistent with Berlyne (1971), we would suggest that aesthetics originates in part from the physical complexity of an object. We propose that four factors affect fractal aesthetic ratings by affecting their perceived complexity: fractal dimension, number of elements in the generator, recursion, and symmetry. Cutting and Garvin (1987) identified these first three in their analysis of judgments about fractals’ perceived complexity, and noted that there is a strong relationship between the number of elements and a method of describing redundancy in patterns, namely Leeuwenberg codes (Leeuwenberg, 1971). Fractal dimension describes the rate at which a pattern increases structure from coarse to fine scales, with higher *D* patterns having more fine structure. Similar to Birkhoff’s and Attneave’s physical complexity descriptions for polygons, the number of elements in the generator of a fractal describes how many elements are introduced at each recursion. Recursion describes the extent to which the pattern repeats itself across scales, whereas symmetry describes this at the level of the overall pattern. Symmetry was not included in Cutting and Garvin (1987) model because they only showed participants radially symmetric exact fractals. Still, studies since Birkhoff (1933) have shown symmetry affects perceptual judgments (Attneave, 1955; Bornstein et al., 1981; Rhodes et al., 1999; Jacobsen and Höfel, 2003), and we have previously shown quite different patterns of visual appeal for statistical fractals (Spehar et al., 2003, 2015; Hagerhall et al., 2004; Spehar and Taylor, 2013) as compared to what we show here for exact fractals. Attneave (1957) provided evidence that symmetry is an important contributor to judgments about polygons’ perceived complexity. We propose that the effect of symmetry does not dissipate with recursion, but acts in complement to it.

Traditionally, theories of aesthetics and perception have used the term symmetry as a way to discuss spatial symmetries, such as mirror and radial symmetry (Birkhoff, 1933; Attneave, 1954, 1955; Berlyne, 1971). Mirror symmetry in faces, or lack thereof, is striking, and contributes to attractiveness ratings (Rhodes et al., 1999). People prefer mirror-symmetric faces (Thornhill and Gangestad, 1999). The preference for mirror-symmetry extends beyond faces. It has been demonstrated that mirror symmetry is one of the most predictive factors when judging if a geometric pattern is beautiful or not (Jacobsen and Höfel, 2003; Jacobsen et al., 2006). Indeed, the preference ratings of two thirds of our participants are reflective of sensitivity to mirror symmetry. But this means that the responses of a surprisingly large subgroup of our participants did not differ as a function of the presence of mirror symmetry. As such, there is a need for further studies to probe the question of whether everyone is swayed by the presence of mirror symmetry in faces and other, non-fractal patterns. Even the results of Jacobsen and Höfel (2003) and Jacobsen et al. (2006) may be driven by individuals who are either more sensitive to mirror symmetry or consistently place greater emphasis on the presence of this symmetry when rating visual appeal. Mirror symmetry is processed differently from infancy (Bornstein et al., 1981) and affects the human and non-human primate brain similarly (Sasaki et al., 2005), so there are evolutionary roots for the neural basis of its perception. These selection pressures may or may not have allowed for variability in sensitivity to such symmetry, but there are individual differences in the extent of its importance for rating the aesthetics of fractals. Spatial symmetry in geometric patterns has been shown to drive activation in brain areas that are involved in perception and evaluative judgment (Sasaki et al., 2005; Jacobsen et al., 2006), yet this too could differ on an individual basis.

We found evidence that these individual differences in preference for mirror symmetry do not extend to radial symmetry. Two germane possibilities may explain this finding. First, the aesthetic value of these, relative to the mirror symmetric fractals, was generally lower, so there may have been a floor effect. Second, we may not have included enough stimuli that were exclusively radially symmetric to have found a similar effect. A third and rather more exotic possibility is that the presence of scaling at a rate near the golden-ratio in the non-symmetric fractals may have a more constant appeal. The golden ratio possesses scale-invariance when repeated, as in the Fibonacci sequence, but lacks spatial symmetry. While several studies have shown that a rectangle with proportions of the golden ratio is most preferred (Fechner, 1871, 1876; Lalo, 1908; Thorndike, 1917), these results have not always been replicated (for a more recent review, see Angier, 1903; Haines and Davies, 1904; Thorndike, 1917; see Green, 1995 or Palmer et al., 2013). Still, Olsen (2006) suggests that approximations of the golden rectangle’s proportions are ubiquitous in nature—observable in phenomena such as shell growth, branching patterns, and the proportions of animals’ bodies, fingers and faces, though others contest such claims (Markowsky, 1992; Livio, 2002). If the golden ratio, or a rough approximation of it, is ubiquitous in natural phenomena’s rate of change across scales, it may be of real importance to aesthetics. Di Dio et al. (2007) disrupted the approximate golden ratio scaling of artworks such as sculptures by manipulating images of them. By doing this at just two scales of measurement, they diminished the participants’ aesthetic response and changed their brains’ patterns of activity (Di Dio et al., 2007). This suggests that repetition of scaling may play a role in aesthetic judgments, perhaps in a way that can compensate for the absence of spatial symmetry.

Such an interpretation is consistent with Leeuwenberg and van der Helm’s (1991) theories and body of work describing a mechanism by which redundancy may be processed by our perceptual systems (Leeuwenberg, 1971, 1978; Leeuwenberg and van der Helm, 1991; van der Helm, 2004). Although there was never an explicit extension of their proposed perceptual mechanisms to aesthetic responses, we will discuss it briefly, in the context of our ability to appreciate fractals’ physical complexity. Inherent to the physical complexity of fractals is redundancy across scales, which we describe in terms of the number of recursions. There is also redundancy in their symmetry. However, the coding theories of Leeuwenberg and van der Helm (1991) fail to explain the patterns of results that we observe. This statement is by no means meant as a criticism of their theories. There is a limited and specific scope of application to which Leeuwenberg and van der Helm (1991) have staked a claim with their theory that elegantly explains how we may process redundancy. Briefly, Leeuwenberg and van der Helm (1991) propose in their more simplistic model that the unique elements of the abstracted code for a pattern, the number of repetitions, and any transformations of that code each contribute a single unit of “load” to a pattern. The code with the lowest load which represents the pattern may be how the human visual system represents a redundant pattern (van der Helm, 2004). Such a code is insensitive to changes in the abstracted code parts. To explain this with an example from our stimulus set, a change in the number of recursions (such as from 10 to 17 for the golden dragon fractals) does not change the load of the abstracted pattern, though there is a striking effect of this change, perceptually, at high levels of fractal dimension (see Figures 4I,L). As such, Leeuwenberg and van der Helm’s (1991) theories make the limited prediction that the visual system will differentiate the golden dragon fractals from the Koch Snowflakes because the abstracted codes of these families of fractals differ, much as they differ in their generator patterns. Their theories do not make predictions about interactions of the abstracted code with changes in the physical units that they represent. As such, the development and testing of theories that predict the roles of other perceptual processes, some of which must be involved in evaluating the differences in physical complexity that vary underneath the same abstracted code, and other psychological processes, some of which must be involved in subjectively evaluating these fractals, will play a crucial role in our understanding of aesthetic responses.

When we look at symmetric, geometric patterns such as exact fractals and find them visually appealing, it may be because they balance interest and comprehensibility (Leder et al., 2004) through the interplay between automatic and active mechanisms (Rentschler et al., 1999; Redies, 2015) as suggested by these recent theories of aesthetics. Individual and group differences may be driving the different patterns of preference for statistical and exact fractals. We have completed studies with statistical fractal stimuli at other universities (Spehar et al., 2003; Hagerhall et al., 2004, 2015; Taylor et al., 2005; Spehar and Taylor, 2013), but have not yet completed a study comparing statistical and exact fractals using a within-subject design. Still, Hagerhall et al. (2015) have shown different trends for alpha-band power of electroencephalography (EEG) recordings during viewing of exact and statistical fractals, so these may be stable differences in response.

It would be a misguided assumption about the homogeneity and stability of human populations over time to say that sample differences could not play a role in the differences in our findings. We only measured a few demographic variables in the present study, and so it could be that our sample is limited along some variable to which we are insensitive. Moreover, individuals from cultures or communities outside of ours may feel differently about exact fractals. Aesthetics can vary with cognitive and cultural factors, which form an important aspect of the Redies (2015) theory. It would be a good test of this aspect of the Redies (2015) model to see whether there are cross-cultural or other individual differences in preference for exact fractals, or whether these are independent of cultural filters. We observed that some individuals prefer lower *D* exact fractals in our first study. While our second sample may have been so small that it voided this population, a clear difference emerged between those who are sensitive to mirror symmetry and those who are not. It could be that by sampling a larger proportion of the population, a different trend in typical preference would be observed because individuals who strongly prefer lower *D* fractals would constitute a greater proportion of the sample. Meanwhile, by exposing the individuals to a broader array of fractal generators in the second study, we may have inadvertently introduced a factor that holds more salience: mirror symmetry. Across the subgroups that differed in their responses to mirror symmetry we observed a consistent effect of dimension on preference that was modulated in magnitude, but not direction, by the presence of symmetry and held, to varying extent, across different levels of recursion.

This interaction between fractal dimension, recursion, and spatial symmetry is important when considering how preference changes across *D* for exact fractals, because it means that there is not universality of preference across exact fractal patterns within individuals. When spatial symmetry was present at high levels of dimension, there was no requirement of a large number of recursions to generate high preference ratings. We discount the alternate interpretation that the interaction is driven by the similarity in the number of recursions of the Koch snowflakes. If that were the case, the preference ratings for those 5 and 6-recursion Koch snowflakes should be at or below the preference ratings for the 10-recursion golden dragons, not equivalent with the 17-recursion golden dragons (see Figure 9 and Table 1). Moreover, preference ratings for the radially symmetric dragons diverged from the golden dragons at higher levels of *D*, suggesting that these patterns are most pleasing when they are symmetric in multiple ways and tend to fill more of the space.

We postulate that the critical factor that allows preference to rise across *D* in this manner is the orderliness of exact fractals. Vitz (1966) showed that random motion traces that filled moderate amounts of space were most preferable, which is consistent with the preference ratings for statistical fractals included in many other studies. A parsimonious account of those studies’ data and our present results is that order is a moderating factor that, when present, preserves interpretability at higher levels of dimension. We have shown that people prefer fractals that fill a greater extent of space (those with higher *D*). Because a greater level of recursion does not affect its base “Leeuwenberg code”, this allows the pattern to approach the maximum space that it can fill at a particular level of dimension while retaining its elegance. Higher recursion has been shown to affect perceived complexity (Cutting and Garvin, 1987), and perceived complexity has been theorized to modulate aesthetic responses (Berlyne, 1971). Given our findings, we theorize that recursion can make up for a lack of spatial symmetry and drive preference upward at peak *D* levels by increasing perceived complexity without affecting the perceived regularity of the pattern. This prediction reflects our interpretation that there is a modulating effect of order on preference for a specific *D* range—increases in the level of recursion should drive up preference for moderately low-*D* statistical fractals and high-*D* exact fractals.

While previous studies of fractal aesthetics have held the level of recursion constant (Spehar et al., 2003; Hagerhall et al., 2004, 2015; Taylor et al., 2005; Spehar and Taylor, 2013), we have shown that it is a variable worthy of further consideration. The interplay between fractal dimension, recursion, and symmetry is an area that warrants further study—especially with respect to physiological correlates by which to explain the basis of these aesthetic responses. Hagerhall et al. (2015) suggest that there is a difference in the processing of exact and statistical fractals. The authors recorded EEG while participants viewed exact and statistical fractal line drawings, and found that waking restfulness (measured by alpha-band power of the EEG signal) was higher for statistical fractals than for exact fractals across a portion of the range of fractal dimension (*D* = 1.1, 1.3, 1.5; Hagerhall et al., 2015).

A putative mechanism that can be tested in future studies is that higher *D* exact fractals are more engaging than their lower *D* counterparts, and this results in higher aesthetic ratings. This analysis could be made more interesting by testing whether individuals who rate low or mid-*D* fractals highest are actually saturating in alpha power response of the EEG signal at that level of *D*, and testing the extent to which alpha power of the EEG signal correlates with preference ratings across the range of *D* for various levels of recursion. These could be used as tests of the Berlyne (1971) arousal model, if it is worthy of further consideration given our results and those of others (Martindale et al., 1990; Vessel and Rubin, 2010; Forsythe et al., 2011). We found individual differences that preclude any conclusions in favor of universality as opposed to mere typicality, even with our limited sample. For those individuals who prefer lower *D* fractals, perhaps there is an interaction between interest and arousal or other aspect of the internal experience that Marin (2015) discusses.

Recent aesthetics models such as those put forth by Cela-Conde et al. (2011), Marin (2015), and Redies (2015) make less specific predictions than the models of the last century (Birkhoff, 1933; Eysenck, 1941; Attneave, 1957; Berlyne, 1971; Boselie and Leeuwenburg, 1985; Cutting and Garvin, 1987), but could be tested with exact and random fractals, as well. Perhaps some individuals find these exact fractals to be beautiful art for which there is perceptual processing of dimension, recursion, and symmetry that is independent of cultural filters. And perhaps to others these are simply not beautiful stimuli—there may be no resonance at the level of perceptual processing. The aesthetic response to fractal patterns could also vary across cultures. Redies (2015) suggests that such patterns are culture-free, a testable prediction. The interplay between perceptual and cognitive processing, modulated by cultural filters (Redies, 2015), could be used as a framework with which we can explain the aesthetics of exact and random fractals. We did not collect sufficient demographic data to test such predictions, but this is an area ripe for future study. Future studies that collect such data may show that the pattern of preference for exact fractals that we observed here is “universal”, as well. Exact fractals provide a new avenue for studying aesthetic responses driven by fractal dimension, recursion and symmetry—features of the patterns that occur in the art of many cultures and in the complex environments of nature.

## Author Contributions

All authors made substantial contributions to the conception and design of the work. CRB created the stimulus library. DRB-G collected the data. AJB analyzed and interpreted the data. AJB and DRB-G drafted the work; AJB revised it with feedback from DRB-G, CRB, RPT, and MES. All authors provided final approval and agree to be accountable for all aspects of the work.

## Conflict of Interest Statement

The authors declare that the research was conducted in the absence of any commercial or financial relationships that could be construed as a potential conflict of interest.
